# Assessing muscular power in older adults: evaluating the predictive capacity of the 30-second chair rise test

**DOI:** 10.3389/fragi.2024.1302574

**Published:** 2024-03-06

**Authors:** Niladri Kumar Mahato, Alexandria Davis, Janet E. Simon, Brian C. Clark

**Affiliations:** ^1^ Ohio Musculoskeletal and Neurological Institute (OMNI), Ohio University, Athens, OH, United States; ^2^ Department of Biomedical Sciences, Ohio University Heritage College of Osteopathic Medicine, Athens, OH, United States; ^3^ College of Osteopathic Medicine, Marian University, Indianapolis, IN, United States; ^4^ Heritage College of Osteopathic Medicine, Ohio University, Athens, OH, United States; ^5^ School of Applied Health and Wellness, Ohio University, Athens, OH, United States

**Keywords:** sarcopenia, muscle strength, muscle power, dynapenia, physical function

## Abstract

**Background:** Timed chair rise tests are frequently used as a substitute for assessing leg muscle strength or power. To determine if timed chair rise tests are an indicator of lower extremity muscle power, we examined the relationship between the repetitions completed in a 30-s chair rise test and the power generated during the test.

**Methods:** Seventy-five individuals participated in this study (n = 30 < 65 years and 45 ≥ 65 years). Participants underwent a 30-s chair rise test while instrumented with a power analyzer. Handgrip strength was also evaluated.

**Results:** The relationship between chair rise repetitions and *average* chair rise power was *R* ^2^ = 0.32 (*p* < 0.001). Chair rise repetitions when regressed on a *total* (i.e., summed) chair rise power, it yielded *R* ^2^ = 0.70 with data from all participants combined (*p* < 0.001). A mediation analysis indicated that anthropometrics partially mediates the relationship between chair rise repetitions and total chair rise power accounting for 2.8%–6.9% of the variance.

**Conclusion:** Our findings indicate that in older adults, the overall performance of chair rises offers limited information about the average power per rise but is more indicative of the cumulative power exerted. Thus, the total number of chair rises in a 30-s test is likely a more comprehensive metric of overall muscular power, reflecting endurance aspects as well. Additionally, we found that personal physical attributes, such as height and weight, partially influence the link between chair rise count and total power, highlighting the importance of factoring in individual body metrics in assessments of muscular performance.

## Introduction

The population of Americans aged 65 and older is projected to almost double by 2060 (US Census Bureau, 2018). This demographic shift coincides with a consistent decline in muscle strength and mass as people age, a condition known as sarcopenia (Frontera et al., 2000). The subset of Americans aged 85 and above, who are highly susceptible to sarcopenia (Cao et al., 2022), is expected to more than double in just the next 15 years. Sarcopenia significantly contributes to limitations in mobility, loss of independence, heightened fall risk, and increased mortality rates (Cruz-Jentoft et al., 2019; Bhasin et al., 2020). For instance, reduced leg extensor strength in older adults is linked to a four-fold increase in the risk for mobility limitations (Manini T. M. et al., 2007). Accordingly, the societal impact of sarcopenia affects healthcare planning for patients, families, caregivers, and insurance providers due to the resultant decline in mobility and autonomy, and thus necessitates a multidimensional and multidisciplinary approach for its assessment and management (Fielding et al., 2011; Cruz-Jentoft et al., 2019; Giovannini et al., 2021). Consequently, sarcopenia has garnered considerable attention in recent years, culminating in its recognition as a disease entity with the assignment of an ICD-10 code in 2016 (Anker et al., 2016).

While there’s no universally agreed-upon definition of sarcopenia, the most recent definition by the European Working Group on Sarcopenia in Older People (EWGSOP2) characterizes it as “muscle failure” (Cruz-Jentoft et al., 2019). This definition marks a shift in how sarcopenia is conceptualized, moving away from low muscle mass to considering low muscle function, such as weakness, as the primary determinant and core aspect of sarcopenia. The EWGSOP2 definition, along with others, suggests timed chair rise tests (such as the 5x chair rise test or the 30-s chair rise test) as a way to approximate leg muscle strength and endurance (Jones et al., 1999; Cruz-Jentoft et al., 2019). These tests are practical for clinical and home settings. Indeed, the 30-s chair rise test has been reported to correlate with lower body muscle strength in older adults living in the community. This supports the idea that it can serve as a surrogate for assessing leg muscle strength (Jones et al., 1999).

Muscular strength refers to the maximum force exerted, while muscle power is the product of force and the velocity of muscle contraction. Muscle power tends to decline earlier and more rapidly with age compared to muscle strength, suggesting that more research should focus on muscle power as a critical outcome in studies related to sarcopenia (Reid and Fielding, 2012). The timed chair rise test, influenced by factors like height and weight, may not be a reliable surrogate for assessing leg muscle power. Few studies have explored the link between timed chair rise tests and muscle power indicators. These studies typically measure power using a leg extension power rig, which might not reflect the specific tasks involved in a chair rise test, leading to potential task specificity issues (Manini et al., 2005; Manini T. et al., 2007). One study found no correlation between a 5x chair rise test and muscle power (Lindemann et al., 2003). However, later research suggested that peak power in a chair rise test often occurs after the fifth rise, indicating a longer test may better evaluate functional power (Smith et al., 2010). Accordingly, in our brief report, we discuss our findings on the correlation between the number of chair rises completed in 30 s and the average and total power exerted during these chair rises.

## Methods

### Study participants

The study involved seventy-five participants ranging in age from 25 to 93 years. Among them, 30 were below 65 years old, and 45 were 65 years old or above. Table 1 presents descriptive statistics detailing the characteristics of these participants. To be eligible, individuals had to be 18 years old. Exclusion criteria included the presence of an implanted pacemaker or any other electronic device, self-reported neurological or neuromuscular diseases, or any condition that, in the opinion of the investigators, could affect participant safety or compromise data quality while performing the specified tasks assessing muscle strength and performance in this study. Approval for the study was obtained from the Ohio University Institutional Review Board.

**TABLE 1:** Descriptive statistics for seventy-five study participants in the study stratified by age: <65 years (n = 30) and ≥65 years (n = 45), and disaggregated by sex. All data are presented as mean ± SD, except for the SPPB data which is shown as median ± quartile ranges.

Group	Age (yr)	Height (cm)	Weight (kg)	BMI (kg/m^2^)	Dominant HGS (kg)	Non-dominant HGS (kg)	SPPB (0–12 score)	30-s chair rise reps (#)	Chair rise power (watts)
Females <65 years (n = 17)	53.4 ± 9.98	173.68 ± 5.77	68.88 ± 19.95	21.24 ± 5.59	24.86 ± 6.94	23.69 ± 6.59*	12.0	20.00 ± 6.00	401.09 ± 86.27
							IQR = 1.0		
Males <65 years (n = 13)	46.85 ± 12.83	175.96 ± 6.39	82.54 ± 13.06	22.04 ± 3.21	42.06 ± 8.28	40.74 ± 10.01	12.0	22.31 ± 4.44	558.72 ± 107.47
							IQR = 0.0		
Females ≥65 years (n = 34)	75.48* ±7.23	160.36 ± 7.22	72.90 ± 15.91	23.24 ± 5.13	17.83 ± 6.36*	17.02 ± 6.86*	10.0	14.16 ± 4.37*	311.32 ± 91.92*
							IQR = 4.50*		
Males ≥65 years (n = 11)	71.09 ± 4.48*	173.36 ± 7.52	85.37 ± 20.17	25.29 ± 5.68	33.66 ± 8.11*	29.15 ± 8.18*	9.0	16.55 ± 4.11*	442.51 ± 78.84*
							IQR = 2.0*		

*Denotes significant difference between the respective sexes for the two age groups, at *p* < 0.05.

HGS, Dominant handgrip strength (mean of three trials).

### Data acquisition

The data included in this report is a segment of a more extensive study. Specifically, we focus on the data related to lower extremity physical performance and handgrip strength in this report.

#### Short physical performance battery (SPPB)

The SPPB was conducted through a series of tests.

1) 4-m Normal Gait Speed Test: Participants completed two trials of walking a 4-m distance at their usual pace, and the time taken for each trial, recorded to the nearest 0.01 s, was averaged.
2) Balance Tests: This evaluated the ability to maintain balance in different positions: side-by-side, semi-tandem and the tandem standing tests. Participants were assessed on their ability to hold each position for 10-s, with the time recorded to the nearest 0.01 s.
3) Five-Times (5x) Chair Rise Test: Participants performed this test on a stable chair with a pan height of 45 cm and no seat padding or arm rests. Chair rises were performed by the participants keeping their arms braced across to their shoulders. The time taken to complete five consecutive chair rises was recorded.

The scores from the gait speed, balance, and chair-rise tests were combined calculate the overall SPPB score (Guralnik et al., 1994).

#### Handgrip strength (HGS)

HGS was measured using a portable Jamar dynamometer (Model 5030 J1; Lafayette Instrument Co.; Lafayette, Indiana), following previously established procedures (Wages et al., 2020). Both dominant and non-dominant handgrips were assessed through alternating three trials on each side, with an option for a fourth trial if the top two trials differed by > 3 kg). The average of the three trials was recorded as the mean HGS for each side.

#### 30-second chair rise test

During the 30-s chair-rise test, participants used the same chair as described previously for the 5x chair test. In this assessment, we recorded the number of repetitions completed during the chair rise test and measured the power output generated throughout the chair rise part of the test. Participants were instructed to perform repeated sit-to-stand movements as fast as possible, ensuring they reached a full standing position before returning to a fully seated position. Throughout the test, participants were connected to a low-friction pulley system attached to their waist. This system was linked to a Tendo Power Analyzer (Tendo Power-Tendo Sports Machines, Trencin, Slovak Republic), allowing us to calculate chair rise power during each rise. We used this data to subsequently calculate the average chair rise power as well as the total (i.e., summed) chair rise power. This method broadly aligns with previous descriptions of quantifying chair rise power (Vincenzo et al., 2018).

### Statistical analysis

The data was divided into two age groups: those <65 years old and those ≥65 years old. Between-group comparisons were conducted using independent t-tests, which were disaggregated by biological sex. For SPPB data, comparisons were made using a Mann Whitney *U* Test due to the non-normal distribution of this variable.

R-squared values were calculated to examine the relationship between the number of repetitions completed during the 30-s chair rise test and the *average* as well as *total* chair rise power achieved in the same duration. This analysis was conducted for the entire sample and separately for the age-stratified groups. We observed a robust association between chair rise repetitions and the total chair rise power. Thus, a mediation analysis was conducted to examine the mediating effect of individual anthropometric measures (height, weight, and BMI) on the relationship between the number of repetitions completed during the 30-s chair rise test and the total chair rise power. A bias-corrected 95% confidence interval of the indirect effects was obtained with 5,000 bootstrapped resamples. A significant indirect effect *via* the mediator between the dependent and independent variables was determined if the 95% confidence interval did not contain zero.

A *post hoc* power analysis revealed that the power for a one-tailed correlation, with alpha set at 0.05, was 0.99 for the entire sample and 0.76 and 0.89 for the younger and older age-stratified groups, respectively. All statistical calculations were performed using the SPSS software (IBM SPSS Statistics, Version 27, Chicago), and significance was determined at two-tailed *p*-value <0.05.

## Results

**FIGURE 1:**
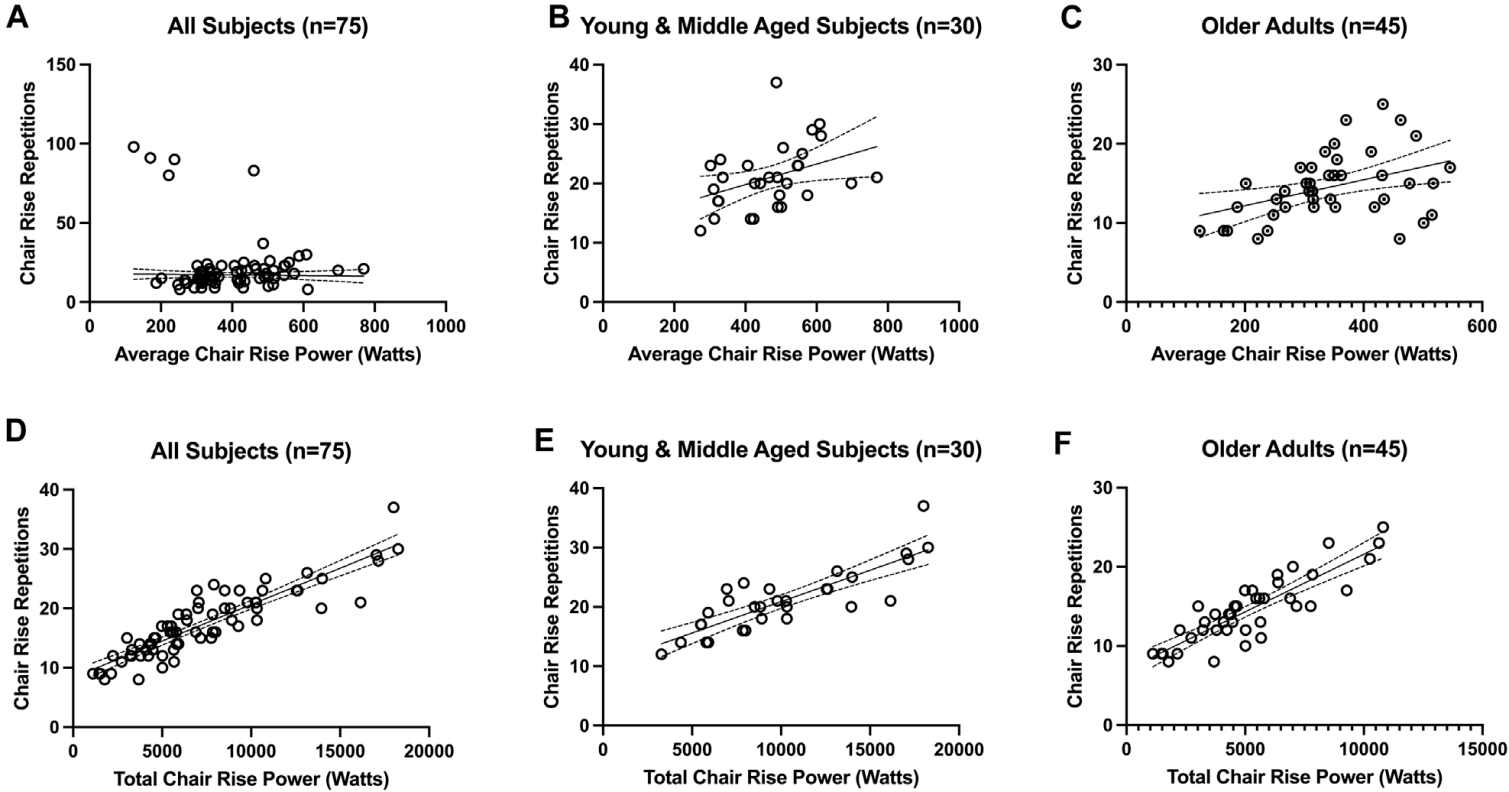
Association between timed chair rise test repetitions and average chair rise power **(A-C)** and total (i.e., summed) chair rise power **(D-F)**. The association between chair rise repetitions and average chair rise power was *R* ^2=^0.32 for the entire sample (i.e., when data from all subjects were combined) (panel A; *p* < 0.001), *R* ^2^ = 0.16 for those under 65 years old (panel B; *p* = 0.031), and *R* ^2^ = 0.17 for those aged ≥65-year (panel C; *p* = 0.006). The association between chair rise repetitions and total chair rise power was *R* ^2=^0.70 for the entire sample (i.e., when data from all subjects were combined) (panel D; *p* < 0.001), *R* ^2^ = 0.70 for those under 65 years old (panel E; *p* < 0.001), and *R* ^2^ = 0.75 for those aged ≥65-year (panel F; *p* < 0.001).

In Table 1, descriptive statistics of the study participants are presented, stratified by age (<65-year and ≥65-year). The association between chair rise repetitions and *average* chair rise power yielded an R-squared value of 0.32 for the entire sample (i.e., when data from all subjects were combined), with a significant *p*-value of <0.001. When considering age groups, the R-squared value was 0.16 for those under 65 years (*p* = 0.031) and 0.17 for those aged 65 or above (*p* = 0.006) (Figures 1A–C). When chair rise repetitions were regressed on *total* (i.e., summed) chair rise power, an *R* ^2^ value of 0.70 was observed as data from all participants were combined (*p* < 0.001). This association was *R* ^2^ of 0.70 for individuals under 65 years (*p* < 0.001) and was *R* ^2^ 0.75 for those aged 65 or above (*p* < 0.001) (Figures 1D–F).

Notably, *average* and *total* chair rise power exhibited a stronger correlation with grip strength compared to chair rise repetitions. Specifically, the R-squared value was 0.64 for *average* chair rise power in relation to both dominant and non-dominant grip strength (*p* < 0.001), while the R-squared values for *total* chair rise power in relation to both dominant and non-dominant grip strength was 0.45 and 0.44, respectively (*p* < 0.001). Conversely, the *R* ^2^ values for the relationship between chair rise repetitions and dominant and non-dominant grip strength were only 0.17 and 0.18, respectively (*p* < 0.002)).

**FIGURE 2:**
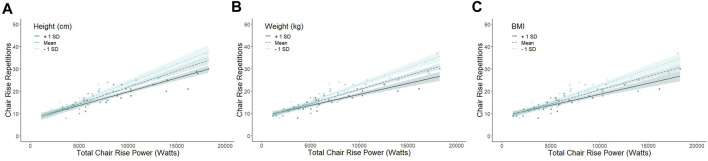
Partial mediation of anthropometric measures on the relationship of chair rise repetitions and chair rise power. The association between chair rise repetitions and chair rise power is partially mediated by height (accounts for 6.9% of the variance between chair rise and power; panel A), weight (accounts for 4.5% of the variance between chair rise and power; panel B), and BMI (accounts for 2.8% of the variance between chair rise and power; panel C). For panel **(A)** height mean = 165.99 cm, +1SD = 175.14 cm, and -1SD = 156.84 cm. For panel **(B)** weight mean = 57.41kg, +1SD = 93.62  kg, and -1SD = 57.41 kg. For panel **(C)** BMI mean = 23.12 kg/m^2^, +1SD = 28.12 kg/m^2^, and -1SD = 18.06 kg/m^2^. Overall, the mediation analyses indicate that individuals who are shorter, weigh less, and have a lower BMI have a stronger and positive relationship between total chair rise power and chair rise repetitions (increase in total chair rise power and chair rise repetitions).

Because we observed a robust association between chair rise repetitions and *total* chair rise power, we conducted a mediation analysis to examine the mediating effect of individual anthropometric measures (height, weight, and BMI) on this relationship. There was partial mediation for height (F_2,69_ = 218.83, *p* < 0.001), weight (F_2,69_ = 181.72, *p* < 0.001), and BMI (F_2,69_ = 160.13, *p* < 0.001). The relationship between chair rise repetitions and *total* chair rise power had 6.9%, 4.5%, and 2.8% of the variance being accounted for by height, weight, and BMI, respectively for each mediation analysis (Figure 2). Overall, the mediation analyses indicate that individuals who are shorter, weigh less, and have a lower BMI have a stronger and positive relationship between total chair rise power and chair rise repetitions (increase in total chair rise power and chair rise repetitions).

## Discussion

Timed chair rise tests are commonly employed as a substitute for assessing leg muscle strength or power in sarcopenia studies. Typically, these tests involve measuring the time taken to complete a set number of repetitions or quantifying the number of chair rise stands within a fixed timeframe. Both methods evaluate absolute chair rise performance. However, it remains uncertain whether absolute chair rise performance directly estimates lower extremity muscle strength, power, or endurance because factors like body anthropometrics can influence this performance (e.g., taller or heavier individuals potentially perform more work than their shorter and lighter counterparts). Moreover, factors such as balance and multi-segment movement coordination might also impact absolute chair rise performance. Previous studies have explored the relationship between the time taken for sit-to-stand chair rise tests and indicators of leg muscle strength and power (Bassey et al., 1992; Skelton et al., 1994; Jones et al., 1999; Bean et al., 2002; Bean et al., 2003; Hardy et al., 2010). However, this evaluated power using a leg extension power rig rather than assessing power during the actual chair rise test, potentially introducing issues related to task specificity (Manini et al., 2005; Manini T. et al., 2007). Consequently, our study investigated the relationship between the number of repetitions completed during a 30-s chair rise test and the chair rise power generated with the same duration.

Our primary finding indicates that in older adults, absolute chair rise performance explains merely 17% of the variance in average chair rise power, indicating that measuring absolute chair rise performance alone (through time or repetitions) is insufficient for accurately assessing average lower extremity muscle power (that is, the power of each repetition, particularly in a 30-s chair stand test). However, in these individuals, absolute chair rise performance is associated with 75% of the variance in total chair rise power. This implies that the count of chair rises within a 30-s period is a good indicator of the overall power exerted during the test. Therefore, the frequency of chair rises observed in this time frame is a better measure of the total power, incorporating aspects of muscular endurance, than the power of each individual chair rise. Muscle performance spans a continuum from generating maximal force against maximal resistance (strength) in a specific exercise to performing repetitions until failure at a certain resistance, which is endurance. Therefore, the 30-s chair rise test can be best understood as a measure of muscle performance within this range, effectively integrating the EWGSOP2 assertions with the current findings on muscle power.

Additionally, we found a more substantial correlation between both the average and total chair rise power with hand grip strength, a recognized biomarker for adverse health outcomes in older adults (Bohannon, 2019), compared to the number of chair rise repetitions. These findings suggest that chair rise power is a more relevant measure for evaluating skeletal muscle performance than merely counting chair rises.

Finally, our research indicates that individual anthropometric factors partially mediate the relationship between the number of chair rises and the total chair rise power, pointing to the influence of physical characteristics on this association. This study faces multiple limitations. Firstly, the absence of power data from the 5x chair rise test barred us from directly comparing the two sit-to-stand paradigms. Secondly, the small sample size limits the generalizability of our findings to the broader population of older adults.

In conclusion, our study supports the notion that the 30-s chair rise test is a meaningful measure of lower extremity muscle power. While it partially reflects muscle endurance due to its association with the total number of chair rises, it offers a more comprehensive metric of overall muscular power. Our findings indicate that the 30-s chair rise test is effective in assessing overall muscular performance, blending strength and endurance aspects. This aligns with EWGSOP2’s view and addresses the gap in research regarding the relationship between timed chair rise tests and direct measures of chair rise power. Furthermore, we note that individual physical characteristics, such as height and weight, play a role in this association, emphasizing the need for considering personal anthropometrics in muscle performance assessments.

## Data Availability

The raw data supporting the conclusion of this article will be made available by the authors, without undue reservation.
